# Trends in acute myocardial infarction-related mortality among adults with type 1 vs. type 2 diabetes in the United States, 1999–2020

**DOI:** 10.3389/fendo.2026.1785552

**Published:** 2026-04-29

**Authors:** Eman Ali, Ayesha Qamar, Ayesha Durrani, Farea Iqbal, Aiman Nasir, Urwah Afzal, Waqas Ullah, Aamir Laghari, Asim Shaikh, Muhammad Sohaib Asghar

**Affiliations:** 1 Dow University of Health Sciences, Karachi, Pakistan; 2 UMass Chan Medical School, Worcester, MA, United States; 3 Department of Cardiology, Owensboro Health, Kentucky, KY, United States; 4 SUNY Upstate Medical University, Syracuse, NY, United States; 5 Department of Internal Medicine, AdventHealth, West Florida, FL, United States

**Keywords:** acute myocadial infarction, CDC WONDER database, diabetes type 2, mortality trajectories, type 1 diabetes mellitus

## Abstract

**Objective:**

Diabetes mellitus (DM) continues to be a major contributor to acute myocardial infarction (AMI)-related mortality. Both T1DM and T2DM are associated with AMI, however, data evaluating the socioeconomic impact and mortality burden of AMI in T1DM remain limited.

**Methods:**

We utilized publicly available, deidentified data from the Centers for Disease Control and Prevention (CDC) Wide-ranging Online Data for Epidemiologic Research (WONDER) database. Using the International Classification of Diseases 10th Revision (ICD-10), we identified diabetic patients with AMI. Age-adjusted mortality rates (AAMRs), standardized per 100,000 population, were stratified by year, sex, ethnicity, urbanization, geographic region, and state. Analyses were conducted using Joinpoint Regression Software version 5.0.2.

**Results:**

Between 1999 and 2020, a total of 203,068 AMI-related deaths occurred among individuals with DM. Of these, 169,973 had T2DM, while 33,095 had T1DM. The overall AAMR was substantially higher in T2DM (4.5) than in T1DM (0.9). Mortality was consistently higher among males (T2DM: 5.0; T1DM: 1.0). Among T2DM patients, the AAMR was highest in Hispanic (5.9), while in T1DM, it was greatest in non-Hispanic (0.9). Across both cohorts, rural areas demonstrated higher mortality (T2DM: 5.9; T1DM: 1.3). T2DM-related mortality peaked in the Western United States (AAMR: 5.3), whereas T1DM-related mortality was highest in the Midwest (AAMR: 1.1). West Virginia showed the highest T2DM mortality (AAMR: 7.9), followed by Iowa, California, Ohio, and Tennessee. For T1DM, Rhode Island reported the highest mortality (AAMR: 1.8), with Tennessee, West Virginia, Arkansas, and North Dakota also ranking in the 90th percentile.

**Conclusion:**

AMI imposes a substantial mortality burden among individuals with DM, especially those with type 2 diabetes mellitus. Marked disparities by sex, ethnicity, region, and urbanization emphasize the need for targeted preventive and healthcare strategies to reduce inequities.

## Graphical Abstract

**Figure:**
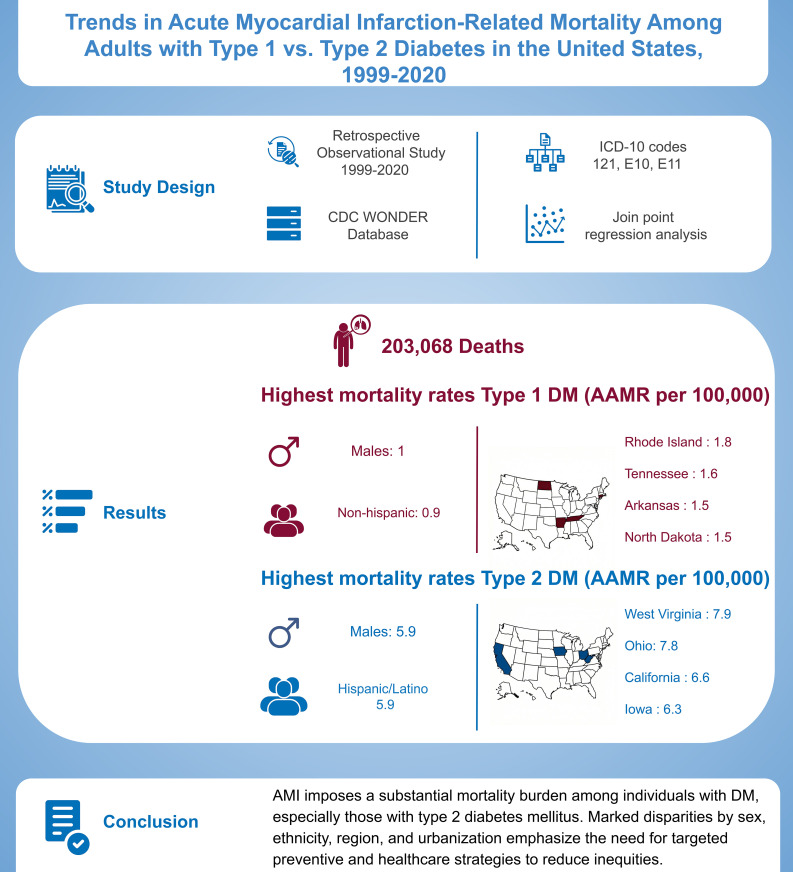

## Introduction

Diabetes mellitus (DM) is an enormous global health concern, with its incidence increasing steadily each year. According to recent estimates from the International Diabetes Federation, approximately 589 million adults aged 20–79 are currently living with diabetes worldwide (1). DM refers to a group of chronic metabolic disorders characterized by persistent hyperglycemia and is associated with significant morbidity and mortality due to a range of long-term complications including peripheral neuropathy, foot ulcers, retinopathy, Charcot joints, and autonomic neuropathy (2). The two main forms of diabetes possess distinct etiologies and disease trajectories. Type 1 diabetes mellitus (T1DM) is an autoimmune condition marked by the destruction of pancreatic β-cells, typically presenting in childhood or adolescence. T1DM is associated with the appearance of anti-islet autoantibodies appearing before the manifestation of clinical disease.The common gene affected is HLA region of chromosome 6 with alleles DR and DQ (3). Type 2 diabetes mellitus (T2DM), the more prevalent form, primarily arises from insulin resistance and genetic predisposition, and is most commonly diagnosed in adults (3, 4). Despite different underlying pathophysiological mechanisms, both forms of diabetes are associated with a markedly increased risk of acute myocardial infarction (AMI) (5).

AMI remains the leading cause of mortality in individuals with diabetes, with approximately one-third of patients hospitalized for an AMI having pre-existing DM (6, 7). T2DM is associated with a fourfold increase in the incidence of AMI and a 2- to 3-fold higher risk of all-cause mortality compared to individuals without diabetes (8). The burden of MI is not limited to T2DM alone. T1DM on the other hand, is a significant risk factor for cardiovascular disease (CVD), driven by a complex interplay of mechanisms such as chronic inflammation, oxidative stress, and endothelial dysfunction. These processes accelerate atherosclerosis, ultimately contributing to conditions like myocardial infarction (MI), heart failure, and ischemic stroke (9, 10). Studies have shown that T1DM is associated with more than a 9-fold increased risk of coronary artery disease (CAD) and a 6-fold higher risk of MI, with female patients experiencing an even greater risk than males (11).

Long-term trends in survival and cardiovascular outcomes for patients with MI have shown significant improvement, however, these patients continue to face a higher risk of mortality and morbidity compared to the general population particularly when additional risk factors such as diabetes are present (12). Previous research has extensively investigated trends in AMI-mortality in T2DM population, with a recently published CDC WONDER database study demonstrating a significant upward trend in AMI-mortality rates among T2DM population from 2014-2020, with comparatively higher mortality in males and hispanic population (13). However, real world insights into AMI-associated mortality in individuals with T1DM remain underexplored. This represents a crucial gap in the literature, as the cardiovascular risk profiles, underlying pathophysiology, and mortality outcomes may differ significantly between T1DM and T2DM populations across different demographic and regional groups.

D ifferentiating between the trajectories of T1DM and T2DM is critical for appropriately interpreting public health trends and assessing healthcare system performance. Combining the two disorders may obscure significant variations in risk, outcomes, and responsiveness to therapies, thus leading to misleading conclusions about overall diabetes care progress. By exploring them separately, policymakers and doctors can better identify gaps in prevention, treatment, and access to care, ensuring that efforts are tailored to the specific issues associated with each health condition. Differences in prevention strategies, long-term care, and population prevalence between T1DM and T2DM play a major role in shaping trends in AMI mortality. T2DM, which is far more common, is strongly influenced by modifiable risk factors such as diet, physical activity, and cardiovascular risk management, making it more responsive to public health interventions and therapeutic advances. On the other hand, T1DM diabetes relies heavily on sustained glycemic control and access to insulin-based therapies, with fewer opportunities for primary prevention. As a result, changes in AMI mortality over time may largely reflect improvements in T2DM care while masking persistent risks in individuals with T1DM. To address this gap, the present study aims to compare trends in AMI-related mortality among adults with T1DM and T2DM in the United States. By delineating these trends, this research seeks to improve risk stratification and guide the development of more effective, tailored prevention strategies for each diabetic subgroup.

## Methods

### Study setting and population

This epidemiological study was conducted in March 2023, utilizing publicly accessible mortality data from Centers for Disease Control and Prevention (CDC) Wide-ranging Online Data for Epidemiologic Research (WONDER) database which is solely based on records from death certificates of US citizens. This data set includes cause of death from death certificates for the 50 states and the District of Columbia and has been previously used in several studies to determine trends in mortality of cardiovascular diseases. We employed Multiple Cause-of-Death Public Use record death certificates to investigate Acute Myocardial Infarction (AMI) as contributing or underlying cause of mortality among adult patients with Type 1 and Type 2 diabetes. The International Statistical Classification of Diseases and Related Health Problems, 10th Revision codes (ICD-10) were applied to identify diabetic patients with AMI: MCD - ICD-10 Codes: I21.0; I21.1; I21.3; I21.4; I21.9; I22.0; I22.1; I22.8; I22.9. Type 2 Diabetics were classified according to following ICD codes: E11.0; E11.1; E11.2; E11.3; E11.4; E11.5; E11.6; E11.7; E11.8; E11.9, whereas Type 1 Diabetics were classified by the following: E10.0; E10.1; E10.2; E10.3; E10.4; E10.5; E10.6; E10.7; E10.8; E10.9.

### Ethics committee

The study utilized publicly available data from the CDC WONDER database, which is de-identified and aggregated to protect individual privacy (14). No personal identifying information was accessed or used in this research, ensuring compliance with confidentiality standards and regulations.

Institutional review board approval was not sought since the data was publicly available, anonymized government data. Additionally, the study was conducted in accordance with the STROBE (Strengthening the Reporting of Observational Studies in Epidemiology) guidelines (15).

### Data extraction

The dataset spanned from the year 1999 to 2020. Adults were defined as those who were 35 years or older at the time of death. Data for population size, year, location of death, demographics, urban-rural classification, region, and states were abstracted. We queried for the following parameters including 1) sex, 2) ethnic background, 3) geographic location, 4) urbanization level, and 5) State. We divided patients into two cohorts i.e. patients with AMI and Type 1 Diabetes versus patients with AMI and Type 2 Diabetes and abstracted their datasets individually to compare mortality trends among both cohorts. Ethnic categories were defined as Hispanic and non-Hispanic due to paucity of data available for racial groups. For geographical stratification Urban-Rural Classification Scheme from the National Center for Health Statistics was employed to categorize the population into urban (large metropolitan area [population ≥1 million], medium/small metropolitan area [population 50,000–999,999]), and rural (non-metropolitan area [population <50,000]) areas (16). Additionally, regions were classified as Northeast, Midwest, South and West according to the US Census Bureau’s classification (17).

### Statistical analysis

We evaluated patterns in AMI related mortality among diabetics by computing age-adjusted mortality rates (AAMRs) per 100,000 population by year, sex, race/ethnicity, state, along with 95% confidence intervals (CIs). When comparing Type 1 Diabetes Mellitus (T1DM) and Type 2 Diabetes Mellitus (T2DM), age-adjustment is crucial because T1DM primarily affects children and young adults, whereas T2DM primarily affects older adults. Additionally, population aging has a significant impact on diabetes trends. Annual Percent Change (APC) and Average Annual Percent Change (AAPC) are used in Joinpoint regression to measure the rate of change over time and to determine when major trend shifts occur. Crude mortality rates were determined by dividing the number of AMI-related deaths by the corresponding U.S. population of that year. AAMRs were calculated by standardizing AMI-related deaths in diabetics to the year 2000 U.S. population (18). Joinpoint Regression Program (Version 5.0.2, National Cancer Institute) was employed to assess temporal changes in mortality (19). Temporal trends in mortality were examined to deduce changes in slope using Joinpoint Regression Program version 4.7.0.0, which models consecutive linear segments on a log scale connected by Joinpoints, where the segments converge. Slopes depicting the change in mortality were considered increasing or decreasing if the estimated slope differed significantly from zero based on 2-tailed t-testing. Statistical significance was set at P ≤ 0.05.

## Results

### Annual trends for AMI-related AAMR among type 1 and type 2 diabetics

Age-adjusted mortality rates (AAMRs) were used to allow comparison between Type 1 and Type 2 diabetes populations with different age distributions, as T2DM predominantly affects older individuals with higher baseline mortality risk. A total of 203,068 AMI-related deaths occurred among diabetics between the years 1999 and 2020. Of which, 169973 deaths were attributed to Type 2 diabetics whereas 33095 deaths occurred in Type 1 diabetics. Throughout the study period, the AAMRs for AMI-related deaths in patients with Type 2 diabetes mellitus (T2DM) had a notable rise, with AAMR 4.2 (95% CI: 4.1-4.3) in 1999 and 6 (95% CI: 5.8-6.1) in 2020. Conversely, the AAMRs in patients with Type 1 diabetes mellitus (T1DM) had a sharp decline with AAMR 2.4 (95% CI: 2.3-2.5) in 1999 and 0.4 (95% CI: 0.4-0.4) in 2020.

Temporal trends in AAMRs were evaluated using Joinpoint regression, where annual percentage change (APC) represents the yearly change in age-adjusted mortality rates over time. The overall AAMRs for AMI-related deaths in T2DM patients were stable between the years 1999 to 2003 (APC: 2.52; 95% CI: -0.06 to 8.84). Following this period, the AAMRs had a significant decline between 2003 and 2014 (APC: -1.85; 95% CI: -4.08 to -1.21). Finally, from 2014 to 2020 a significant increase in AAMRs was observed (APC: 6.68; 95`% CI: 5.05 to 8.97). In contrast, T1DM patients exhibited a significant drop in AAMRs between 1999 and 2001 (APC: -13.43; 95% CI: -15.24 to -10.58). The AAMRs declined further significantly from 2001 to 2012 (APC: -10.08; 95% CI: -11.16 to -3.07) and then remained stable between the 2012 to 2020 period (APC: -4.52; 95% CI: -8.98 to 3.59). Demographic characteristics, including the number of deaths, AAMRs, APCs, and AAPCs among T2DM and T1DM patients with AMI-related mortality, have been highlighted in **Table 1**, **Table 2** and **Supplementary Table 1**.

**Table 1:** Demographic characteristics of deaths due to AMI and Diabetes among adults in the USA from 1999 to 2020.

	Type 2 diabetes		Type 1 diabetes	
Variable	Deaths	AAMRs	Deaths	AAMRs
Overall	169973	4.5	33095	0.9
Sex				
Male	96190	5.9	17504	1
Female	73783	3.4	15591	0.7
Race				
Hispanic	16383	5.9	2,084	0.7
Non-Hispanic	153261	4.4	30966	0.9
Non-stated	329	-	61	-
Urban/Rural				
Urban	130598	4.2	24567	0.8
Rural	39375	5.9	8528	1.3
Census Region				
Northeast	23399	3.1	6377	0.9
Midwest	44153	5.2	9260	1.1
South	59080	4.3	12238	0.9
West	43341	5.3	5220	0.6

**Table 2:** Annual percentage changes (APCs) and average annual percentage changes (AAPCs) in AMI-related mortality rate among diabetic adults in the USA from 1999 to 2020.

Variable	Type 2 diabetes						Type 1 diabetes					
Overall	Segment	Lower endpoint	Upper endpoint	APC	Lower CI	Upper CI	Segment	Lower endpoint	Upper endpoint	APC	Lower CI	Upper CI
	1	1999	2003	2.522	-0.0618	8.8476	1	1999	2001	-13.4267*	-15.2353	-10.5762
	2	2003	2014	-1.8542*	-4.0787	-1.21	2	2001	2012	-10.0780*	-11.1639	-3.069
	3	2014	2020	6.6776*	5.0527	8.973	3	2012	2020	-4.5188	-8.9806	3.5875
Race												
Hispanic or Latino	1	1999	2014	0.0159	-7.922	14.5646	1	1999	2001	-24.2985*	-28.4365	-16.0892
Hispanic or Latino	2	2014	2018	4.5151	-5.0259	7.5198	2	2001	2020	-8.5097*	-9.6716	-7.5854
Hispanic or Latino	3	2018	2020	19.1540*	9.0312	27.4168						
Not Hispanic or Latino	1	1999	2003	2.7791*	0.3856	7.9626	1	1999	2007	-11.4838*	-12.864	-10.6661
Not Hispanic or Latino	2	2003	2014	-2.0826*	-3.5878	-1.479	2	2007	2020	-6.8468*	-8.2135	-4.9954
Not Hispanic or Latino	3	2014	2020	6.2505*	4.671	8.4874						
Gender												
Female	1	1999	2003	2.1536	-0.5759	8.4598	1	1999	2007	-12.5700*	-16.0461	-11.4805
Female	2	2003	2014	-2.7590*	-5.2711	-2.054	2	2007	2020	-8.0283*	-10.1763	-1.7362
Female	3	2014	2020	5.8346*	3.8829	8.6846						
Male	1	1999	2003	2.6915	-0.0264	9.8448	1	1999	2008	-10.4473*	-11.4542	-9.8331
Male	2	2003	2014	-1.4209*	-4.5144	-0.7068	2	2008	2020	-6.0464*	-7.2897	-4.3362
Male	3	2014	2020	7.2038*	5.5432	9.7304						
Urbanization												
Urban	1	1999	2004	1.5083	-0.6705	8.5014	1	1999	2008	-12.2952*	-14.3261	-11.3173
Urban	2	2004	2014	-1.6929	-5.9128	2.3853	2	2008	2020	-5.9377*	-8.3095	-1.6776
Urban	3	2014	2020	6.9959*	5.0697	10.4291						
Rural	1	1999	2002	5.9234*	2.654	12.2239	1	1999	2009	-9.3113*	-11.5382	-8.5905
Rural	2	2002	2014	-2.4325*	-3.1505	-1.9639	2	2009	2020	-5.6827*	-7.5079	-0.1227
Rural	3	2014	2020	6.4496*	5.0549	8.5291						
Census Region												
Census Region 1: Northeast	1	1999	2003	-0.3678	-2.4093	4.0732	1	1999	2007	-12.1439*	-15.238	-11.0205
Census Region 1: Northeast	2	2003	2013	-4.3667*	-8.2038	-3.7544	2	2007	2020	-7.1226*	-9.2119	-2.6975
Census Region 1: Northeast	3	2013	2018	1.4451	-3.095	5.5108						
Census Region 1: Northeast	4	2018	2020	17.5640*	9.8607	23.469						
Census Region 2: Midwest	1	1999	2005	0.9622	-0.5498	4.1166	1	1999	2008	-12.1759*	-14.2114	-11.1174
Census Region 2: Midwest	2	2005	2013	-3.9669*	-8.122	-2.7644	2	2008	2020	-6.0868*	-8.4568	-1.5805
Census Region 2: Midwest	3	2013	2020	5.1297*	3.5256	7.5801						
Census Region 3: South	1	1999	2003	3.4880*	0.2683	10.5638	1	1999	2007	-11.0205*	-13.5273	-10.1786
Census Region 3: South	2	2003	2014	-2.9878*	-5.3962	-2.172	2	2007	2020	-7.9716*	-9.4409	-2.5487
Census Region 3: South	3	2014	2020	6.3804*	4.1842	9.9975						
Census Region 4: West	1	1999	2008	4.4236*	2.5656	6.3152	1	1999	2011	-8.8648*	-12.9912	-7.806
Census Region 4: West	2	2008	2011	-2.2598	-16.6928	14.6737	2	2011	2020	-2.5238	-6.4186	15.2112
Census Region 4: West	3	2011	2020	6.3366*	5.1365	7.5503						

* represents significant mortality trends observed in respective years.

### Mortality trends stratified by sex

**Figure 1:**
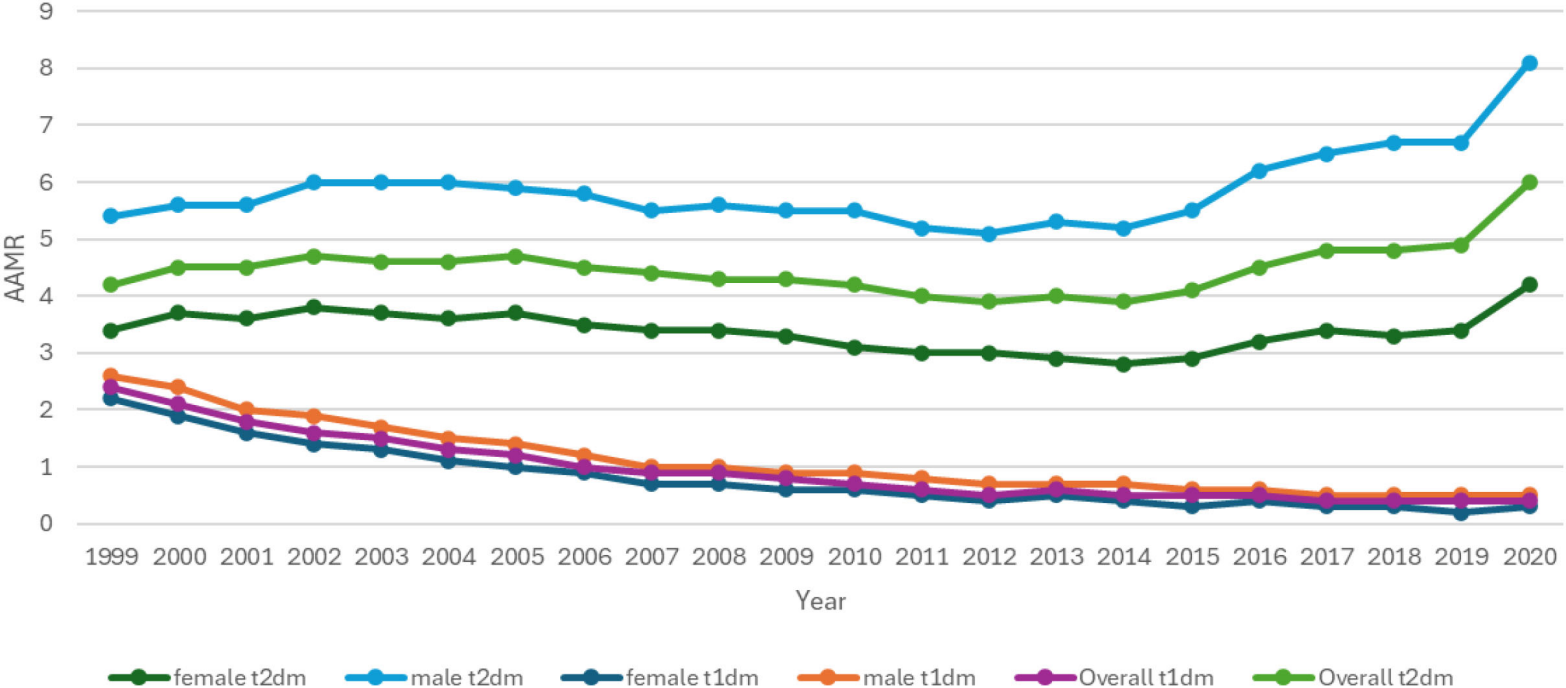
AMI-related mortality trends in diabetic adult population overall and stratified for sex in 1999–2020.

**Figure 2:**
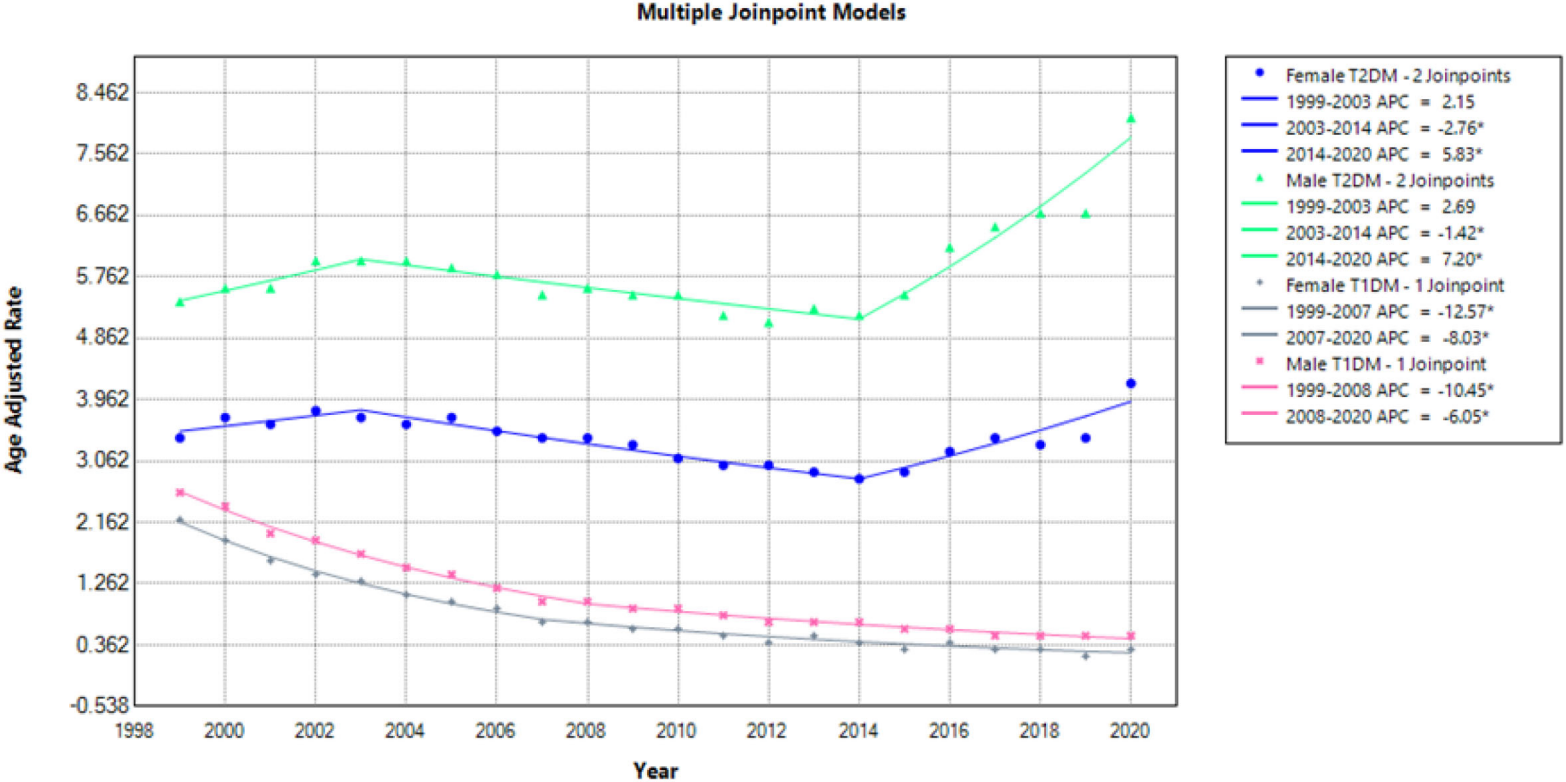
Annual percentage change (APCs) for AMI-related mortality in diabetic adults compared for both sex groups.

Stratification by sex revealed a total of 96,190 deaths in males with T2DM and 17,504 deaths in males with T1DM. Females with T2DM and T1DM had 73783 and 15591 deaths respectively (**Table 1**; **Supplementary Table 2**). Across the study period, males consistently exhibited higher mortality rates compared to females (**Figures 1**, **2****).** The overall AAMR among men was 5.9 for T2DM and 1.0 for T1DM. In contrast, females had lower overall AAMRs, with 3.4 for T2DM and 0.7 for T1DM.

Men with T2DM had the highest rate of mortality from the start till the end of the study period. The AAMR for men with T2DM in 1999 was 5.4 (95% CI: 5.2-5.6) which remained steady till 2003 and then had a significant decline reaching an AAMR of 5.2 in 2014 (APC: -1.42; 95% CI: - 4.51 to - 0.71). From 2014 to 2020, the mortality rate increased significantly reaching AAMR of 8.1 (APC: 7.20; 95% CI: 5.54 to 9.73). Females with T2DM had a higher MI-related mortality compared to females with T1DM but lower than males with T2DM. The AAMR in females with T2DM in 1999 was 3.4 (95% CI: 3.2-3.5). It remained steady till 2003 after which there was a significant decline with an AAMR of 2.8 in 2014 (APC: -2.76; 95% CI: - 5.27 to - 2.05). From 2014 to 2020 the AAMR significantly increased to 4.2 (APC: 5.83; 95% CI: 3.88 to 8.68) (**Figures 1**, **2**; **Supplementary Table 2**).

T1DM patients, both male and female, had a consistent decrease in mortality rates. The AAMR in men with T1DM in 1999 was 3.0 (95% CI: 2.5-2.7) which had a significant decline in 2008 leading to an AAMR of 1.0 (APC: -10.45; 95% CI: - 11.45 to - 9.83). From 2008 to 2020 there was again a noticeable decline (APC: -6.05; 95% CI: - 7.29 to - 4.34). In T1DM females, there was a significant decline from 1999 onwards with an AAMR of 2.0 (95% CI: 2.1-2.3) in 1999 reaching 1.0 in 2007 (APC: -12.57; 95% CI: - 16.0 to - 11.5) and AAMR of 0.0 in 2020 (APC: - 8.03; 95% CI: -10.18 to -1.74) (**Figures 1**, **2**, **Supplementary Table 2**).

### Mortality trends stratified by urbanization

**Figure 3:**
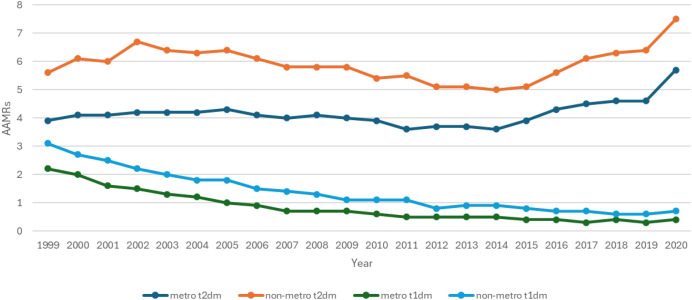
AMI-related mortality trends in diabetic adult population and stratified for urbanization in 1999–2020.

**Figure 4:**
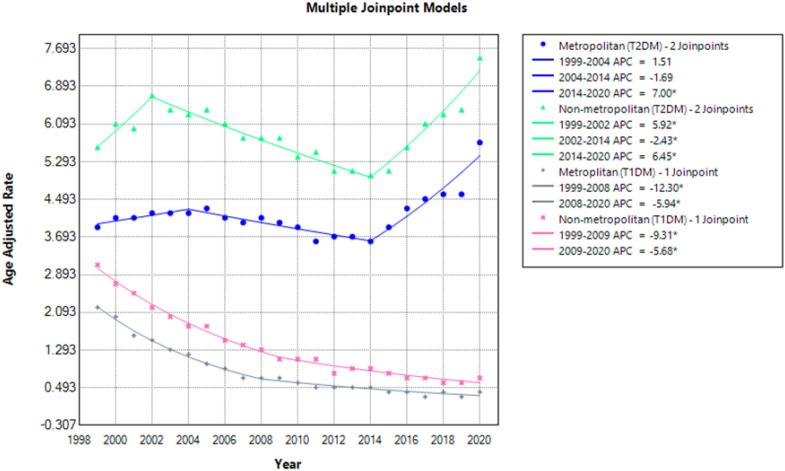
Annual percentage change (APCs) for AMI-related mortality in diabetic adults stratified by urbanization.

Stratification by urban-rural classification revealed rural areas to have the highest mortality rate in both diabetic groups (**Figures 3**, **4**). MI-related deaths due to Type 2 diabetes remained higher than Type 1 diabetes. T2DM in rural areas during 1999 had an AAMR of 5.6 (95% CI: 5.4-5.9) which significantly increased to 6.7 in 2002 (APC: 5.92; 95% CI: 2.65 to 12.2), decreased considerably to 5.0 in 2014 (APC: -2.43; 95% CI: - 3.15 to - 1.96) and then again had a significant rise to 7.5 in 2020 (APC: 6.45; 95% CI: 5.05 to 8.53). Urban areas in T2DM patients showed an AAMR of 3.9 (95% CI: 3.8-4.0) in 1999 which remained steady until 2014 and then got significantly elevated to 5.7 in 2020 (APC: 6.99; 95% CI: 5.07 to 10.43). A notable increase in T2DM rates was observed in both urban and rural populations from 2014 to 2020.

T1DM showed a similar decreasing trend for both groups. Rural areas stood with an AAMR of 3.1 (95% CI: 2.9-3.3) in 1999. It declined significantly to 1.1 in 2009 (APC: -9.31; 95% CI: - 11.54 to - 8.59) and decreased to 0.7 in 2020 (APC: -5.68; 95% CI: - 7.51 to - 0.12). T1DM patients in urban areas had a significant drop in AAMR from 2.2 (95% CI: 2.1-2.3) in 1999 to 0.7 in 2008 (APC: -12.30; 95% CI: - 14.3 to - 11.3) which then again had a significant drop to 0.4 in 2020 (APC: -5.94; 95% CI: - 8.31 to - 1.68) (**Supplementary Table 3****).**

### Mortality trends stratified by geographical location

**Figure 5:**
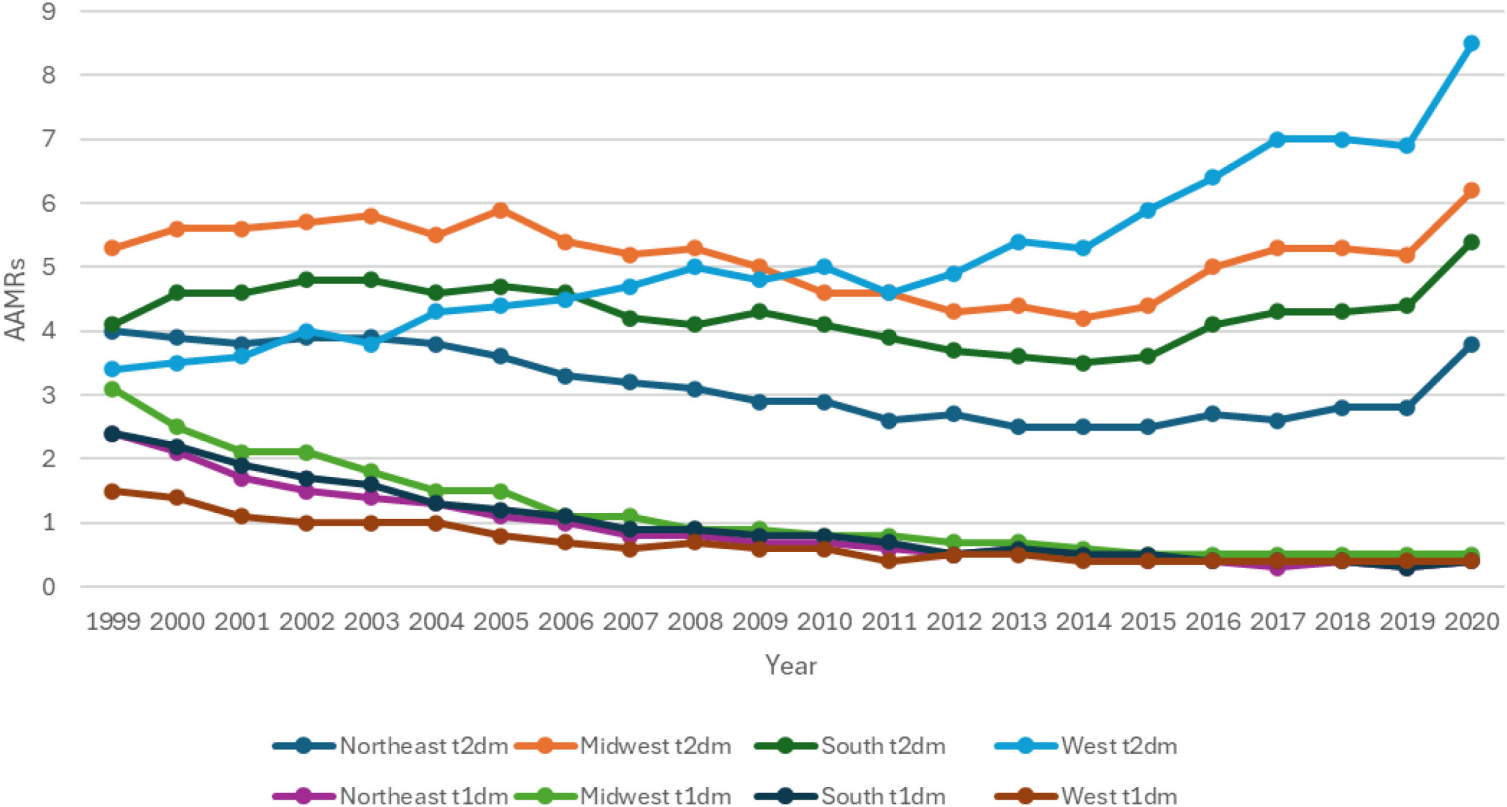
AMI-related mortality trends in diabetic adult population and stratified for geographic location in 1999–2020.

**Figure 6:**
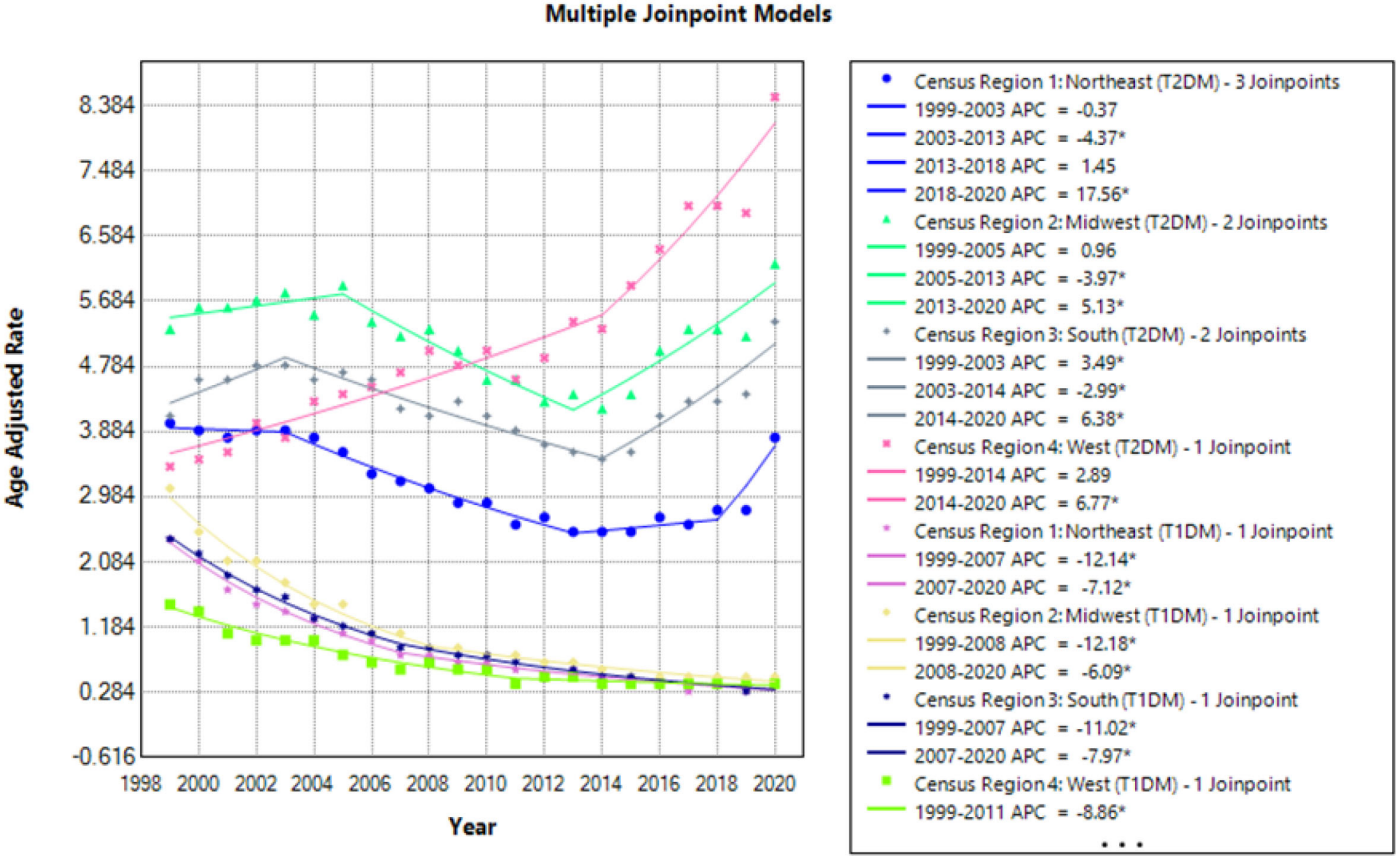
Annual percentage change (APCs) for AMI-related mortality in diabetic adults stratified by geographic location. * represents significant mortality trends observed in respective years.

When stratified for geographical location, Type 2 diabetics had highest mortality was observed in the Western region (AAMR: 5.3) followed by Midwestern (AAMR: 5.2), Southern (AAMR: 4.3), and Northeastern (AAMR: 3.1) regions. In contrast Type 1 diabetics had the highest mortality in the Midwestern (AAMR: 1.1) region followed by Northeastern (AAMR: 0.9), Southern (AAMR: 0.9) and Western region (AAMR: 0.6) (**Figures 5**, **6**; **Supplementary Table 4**).

T2DM patients showed broadly similar AAMR trends across most U.S. regions, with the exception of the West region. The overall trend in age-adjusted mortality rates (AAMRs) for T2DM demonstrated a three-phase pattern across most U.S. regions. Initially, from 1999 to the early 2000s, AAMRs were either stable or exhibited a modest increase. This was followed by a notable decline across several regions from the early 2000s to around 2014. From 2014 onwards, a reversal of this trend was observed, with a significant rise in AAMRs seen across all regions. The Southern region (Region 3) exhibited a significant rise in AAMR during the initial 1999–2003 period (APC: 3.49; 95% CI: 0.27 to 10.56), before aligning with the overall national trend of a mid-period decline followed by a subsequent increase from 2014 onward. The most notable rise during the 2018–2020 period was seen in the Northeast region (Region 1) (APC: 17.56; 95% CI: 9.86 to 23.47). Conversely, the West region (Region 4) had a steady uprise in AAMR from 1999 to 2014 with no periods of decline and then showed considerable elevation in AAMRs until 2020. T1DM rates displayed a consistent decline across all regions, with the most pronounced decrease in the Midwest region (Region 2) during the 1999–2008 period (APC: -12.18; 95% CI: - 14.2 to - 11.1).

A general trend of steady AAMRs was observed followed by a rapid decline during the 2003 to 2014 period and then an eventual rise in mortality rates from 2014 to 2020.

### Mortality trends stratified by ethnicity

**Figure 7:**
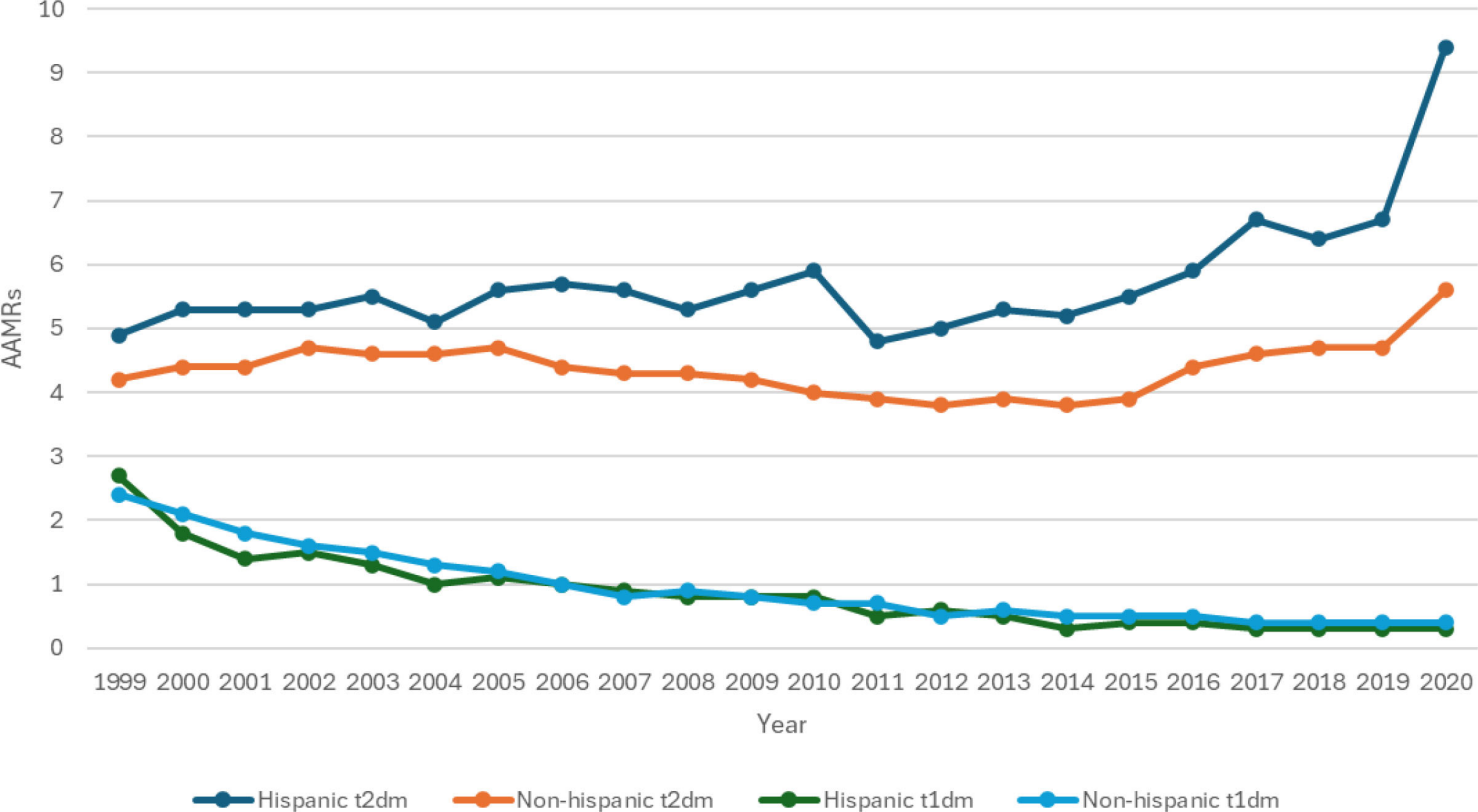
AMI-related mortality trends in diabetic adult population and stratified for ethnicity in 1999–2020.

**Figure 8:**
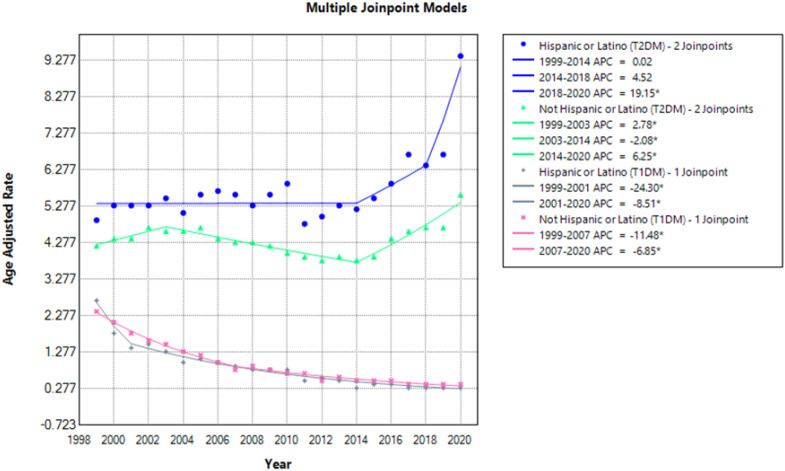
Annual percentage change (APCs) for AMI-related mortality in diabetic adults stratified by ethnicity in 1999–2020.

When stratified by race/ethnicity, mortality rates were highest for T2DM patients in the Hispanic. Both the Hispanic and Non-Hispanic showed similar steady patterns in AAMR during the periods 1999–2014 after which they increased. A striking increase in T2DM rates was observed in the Hispanic population from 2018 to 2020 (APC: 19.15; 95% CI: 9.03 to 27.4) significantly exceeding the rate of increase in the non-Hispanic population during the same period. For T1DM patients however, the AAMR decreased for both groups with a significant rate of decrease seen in the Hispanic group during the 1999–2001 period (APC: -24.30; 95% CI: - 28.4 to - 16.1) (**Figures 7**, **8**; **Supplementary Table 5****).**

### Mortality trends stratified by state

When stratified state-wise, patients with T2DM were noted to have higher mortality rates compared to patients with T1DM. Among patients with T2DM, the lowest mortality rate was observed in the state of Nevada with an AAMR of 1.4 (95% CI: 1.2-1.5) whereas the highest rate was observed in West Virginia with an AAMR of 7.9 (95% CI: 7.6-8.3). States falling within the top 90th percentile of AAMRs for T2DM included Iowa, California, Ohio, Tennessee, West Virginia. In contrast, states within the bottom 10th percentile comprised Nevada, Connecticut, Massachusetts, Louisiana, and Georgia, each exhibiting mortality rates less than half of those in the highest-burden states.

**Figure 9:**
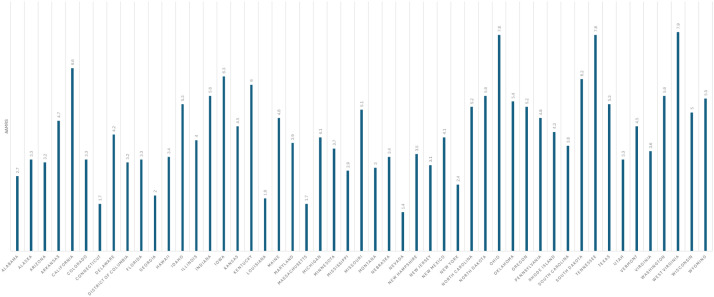
AMI-related mortality trends in type 2 diabetic adult population and stratified by state in 1999–2020.

**Figure 10:**
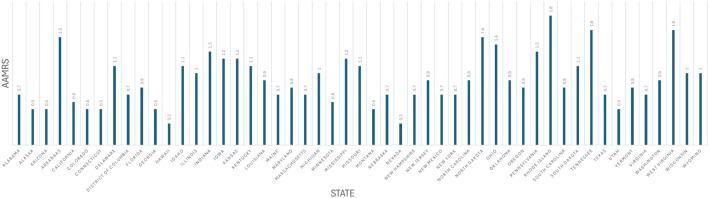
AMI-related mortality trends in type 1 diabetic adult population and stratified by state in 1999–2020.

There was minimal variation in mortality rates seen across the T1DM group. Hawaii and Nevada were among states with the lowest mortality rate (Hawaii: AAMR: 0.3; 95% CI: 0.2-0.4; Nevada: AAMR: 0.2-0.4) whereas Rhode Island displayed the highest mortality rate of 1.8 (95% CI: 1.6-2.1). States within the top 90th percentile comprised Rhode Island, Tennessee, West Virginia, Arkansas, and North Dakota, reflecting more than a fivefold difference in mortality compared to the lowest-burden states. States falling within the bottom 10th percentile included Hawaii, Nevada, Alaska, Arizona, and Colorado. Several other states (eg, Georgia, Connecticut, Montana, Utah) had identical rates at the 10th percentile cutoff (**Figures 9**, **10**; **Supplementary Table 6****).**

## Discussion

DM is a well-established chronic disease that complicates the clinical course of AMI and confers a markedly higher risk of adverse outcomes and mortality than in non-diabetic patients (20). This study analyzed mortality trends in the U.S. population from 1999 to 2020 and identified 203,068 deaths due to AMI among adults (aged ≥35 years) with diabetes, including 169,973 in T2DM and 33,095 in T1DM. The temporal trajectories diverged sharply by diabetes type. Among adults with T2DM, AAMRs rose modestly in the early 2000s, declined substantially from 2003 to 2014, and then reversed with a steep rise between 2014 and 2020. By contrast, mortality among adults with T1DM declined steadily throughout the study period, with AAMRs falling from 2.4 in 1999 to 0.4 in 2020. Mortality remained consistently higher among men than women across both diabetes types throughout the study period. Among racial groups, Hispanic adults with T2DM had the highest AAMRs, particularly between 2018 and 2020, while non-Hispanic adults had persistently lower rates. Significant geographical variation was also observed, for T2DM West Virginia reported the highest state-level AAMR and Nevada the lowest, whereas T1DM mortality was highest for Rhode Island and lowest in Hawaii and Nevada. Regionally, T2DM mortality was highest in the West and lowest in the Northeast, following a three-phase trend of early stability, mid-period decline, and post-2014 rise; T1DM mortality declined steadily across all regions, most notably in the Midwest. Additionally, a persistent urban-rural divide was evident, with rural areas consistently demonstrating higher mortality rates throughout the study period.

The decline in AAMRs from the early 2000s to 2014 is likely to reflect the cumulative impact of improved cardiovascular care and diabetes management. Widespread adoption of statins, antihypertensive agents, and renin-angiotensin system inhibitors, together with improvements in reperfusion strategies and standardized AMI protocols, improved survival outcomes in both T1DM and T2DM populations (21). Simultaneously, advances in diabetes management (including novel insulin analogs, continuous glucose monitoring, and structured risk factor control) helped reduce vascular complications (22), while public health efforts promoting smoking cessation and healthier lifestyles reinforced these gains (23). However, from 2014 onwards, these gains plateaued and reversed, may reflect the growing prevalence of obesity, metabolic syndrome, and early-onset T2DM (24, 25), alongside stagnation in blood pressure and lipid control (26). Mortality patterns were also influenced by disparities in healthcare quality and access, particularly where therapeutic advances were unevenly distributed. The early phase of the COVID-19 pandemic may have contributed to these trends through disruptions in healthcare delivery and differential impact on individuals with cardiometabolic disease, including screening and chronic disease management (27, 28).

Men consistently exhibited higher AAMRs than women across both T1DM and T2DM, a disparity that may stem from both biological and sociocultural factors. Hormonal pathways are central with estrogen exerting vasoprotective, anti-inflammatory and endothelial-stabilizing effects (29), whereas testosterone is linked to impaired vascular repair and adverse immune modulation (30). Men with diabetes also show greater platelet reactivity, heightened thrombotic risk, and a predominance of obstructive coronary atherosclerosis, amplifying AMI mortality, while women more often present with microvascular dysfunction, which carries lower acute mortality (31, 32). These biological differences, compounded by higher smoking prevalence, delayed care seeking, and poor adherence to preventive therapy among men (33), may contribute to higher mortality despite wider use of reperfusion therapies (31).

Profound racial and ethnic disparities were evident across both T1DM and T2DM. Hispanic adults experienced the greatest mortality burden in T2DM, with a striking acceleration between 2018 and 2020 during the COVID-19 pandemics, whereas non-Hispanic adults exhibited persistently elevated but comparatively lower rates across the two-decade period in both diabetes types, a difference reflected by lower prevalence of obesity and metabolic syndrome, earlier diagnosis, and broader access to cardioprotective therapies (34, 35). This aligns with the prior evidence showing higher AMI-related mortality among Hispanic adults due to intersecting factors such as clinical risk, delayed presentation, and limited treatment access (36). Socioeconomic factors further contribute to these disparities, with uninsured status, lower education, and limited income contributing to excess mortality-risk (37). The post-2018 rise in mortality among Hispanic adults may reflect their increased vulnerability during the crisis, potentially due to occupational exposure, language barriers, or healthcare access challenges (38).

Geographical and regional disparities further emphasized the impact of structural determinants on health outcomes. Among adults with T2DM, states such as West Virginia reported the highest AMI mortality, whereas Nevada had the lowest, likely reflecting substantial differences in healthcare infrastructure, insurance coverage, and chronic disease prevalence (39). In T1DM, mortality was highest in Rhode Island and lowest in Hawaii and Nevada, though absolute rates remained substantially lower than T2DM. Socioeconomic factors, educational attainment, and health literacy likely influence these disparities by reducing engagement with preventive care, adherence to therapy, and timely recognition of acute events (40, 41). At the regional level, T2DM mortality was highest in West, where persistently elevated AAMRs without the mid-period decline observed elsewhere may reflect enduring gaps in preventive uptake, risk factor control, and access to timely cardiovascular interventions (42). By contrast, the Northeast consistently recorded the lower rates until a sharp escalation between 2018 and 2020, likely reflecting the compounded effect of COVID-19, which magnified existing vulnerabilities and disrupted chronic disease management (43). The steady decline in T1DM mortality across all regions, most pronounced in the Midwest, suggests that advances in insulin therapy, continuous glucose monitoring, and structured diabetes care have been disseminated more equitably, mitigating regional variation (44).

The urban-rural mortality divide remained persistent throughout the study period. Rural areas often lack timely access to advanced care and diagnostic services essential for effective MI and diabetes management (45, 46). Structural limitations such as healthcare workforce shortages and higher poverty rates may exacerbate these challenges (47). These findings support the role of AMI-diabetes mortality as a bellwether for systemic healthcare inequities, particularly during public health crises.

## Limitations

Despite the strengths of this large, population-based analysis, several limitations should be noted. First, reliance on death certificate data from the CDC WONDER database introduces the potential for misclassification bias, including between T1DM and T2DM, and is subject to coding errors, selection bias, and incomplete or improper reporting, particularly in distinguishing between underlying and contributing causes of death. Variability in diagnostic criteria and coding practices for MI and diabetes may affect the accuracy of cause-specific mortality estimates. Second, the absence of individual-level data prevents adjustment for diabetes duration, glycemic control, treatment modalities, comorbidities, socioeconomic status, healthcare utilization. Third, small numbers in some T1DM subgroups, particularly in sex-stratified analyses, may produce unstable mortality rates and should be interpreted cautiously. These fluctuations may reflect random variation rather than true temporal changes in mortality patterns. Fourth, regional aggregation may obscure within-region heterogeneity and changes in clinical guidelines and coding standards over the study period may also have influenced the observed trends. Fifthly, binary stratification (Hispanic vs. non-Hispanic) was necessitated by reporting consistency and small-cell suppression protocols within the CDC WONDER database, which limited our ability to reliably analyze more granular race/ethnicity categories. Finally, while inclusion of 2020 data enhances relevance, the full impact of the COVID-19 pandemic on mortality, particularly in relation to comorbid chronic diseases, remains incompletely captured. Future studies should utilize individual-level data to elucidate the complex drivers of geographic and temporal mortality variation.

## Conclusion

AMI related mortality among adults with DM in the United States remains unacceptably high and deeply uneven. Type 2 diabetes accounts for the vast majority of deaths, with striking state-level variation reflecting longstanding disparities in health system capacity, socioeconomic vulnerability, and access to preventive and acute cardiovascular care. Despite the decline in type 1 diabetes, geographic gaps persist. The sustained burden in the western United States and the abrupt rise in mortality in the northeast after 2018 may reflect the combined effects of chronic underinvestment and the disruptive impact of the COVID-19 pandemic. Persistently higher mortality in rural settings further highlights structural barriers to timely access to life-saving interventions. These findings underscore the need for coordinated national strategies that strengthen regional health infrastructure, expand equitable access to high-quality cardiovascular and diabetes care, and prioritise structural reforms to address the social determinants driving excess mortality.

## Data Availability

The original contributions presented in the study are included in the article/**Supplementary Material**. Further inquiries can be directed to the corresponding author.
