# Supplementary material for: Trends in acute myocardial infarction-related mortality among adults with type 1 vs. type 2 diabetes in the United States, 1999–2020
Source: Front Endocrinol (Lausanne). 2026 Apr 29;17:1785552. doi: 10.3389/fendo.2026.1785552 (PMC13167565; doi:10.3389/fendo.2026.1785552)
Supplement: Supplementary file 1 [file Table1.docx]

Supplementary Tables

Table 1: Overall AAMRS and 95% Confidence Intervals from 1999-2020.

|  | **Type 2 Diabetes** | | | **Type 1 Diabetes** | | |
| --- | --- | --- | --- | --- | --- | --- |
| **Year** | **Age Adjusted Rate** | **Age Adjusted Rate Lower 95% CI** | **Age Adjusted Rate Upper 95% CI** | **Age Adjusted Rate** | **Age Adjusted Rate Lower 95% CI** | **Age Adjusted Rate Upper 95% CI** |
| 1999 | 4.2 | 4.1 | 4.3 | 2.4 | 2.3 | 2.5 |
| 2000 | 4.5 | 4.4 | 4.6 | 2.1 | 2 | 2.2 |
| 2001 | 4.5 | 4.4 | 4.6 | 1.8 | 1.7 | 1.8 |
| 2002 | 4.7 | 4.6 | 4.8 | 1.6 | 1.5 | 1.7 |
| 2003 | 4.6 | 4.5 | 4.7 | 1.5 | 1.4 | 1.5 |
| 2004 | 4.6 | 4.5 | 4.7 | 1.3 | 1.2 | 1.3 |
| 2005 | 4.7 | 4.6 | 4.8 | 1.2 | 1.1 | 1.2 |
| 2006 | 4.5 | 4.4 | 4.6 | 1 | 1 | 1.1 |
| 2007 | 4.4 | 4.3 | 4.5 | 0.9 | 0.8 | 0.9 |
| 2008 | 4.3 | 4.2 | 4.4 | 0.9 | 0.8 | 0.9 |
| 2009 | 4.3 | 4.2 | 4.4 | 0.8 | 0.7 | 0.8 |
| 2010 | 4.2 | 4.1 | 4.3 | 0.7 | 0.7 | 0.8 |
| 2011 | 4 | 3.9 | 4.1 | 0.6 | 0.6 | 0.7 |
| 2012 | 3.9 | 3.8 | 4 | 0.5 | 0.5 | 0.6 |
| 2013 | 4 | 3.9 | 4.1 | 0.6 | 0.5 | 0.6 |
| 2014 | 3.9 | 3.8 | 4 | 0.5 | 0.5 | 0.5 |
| 2015 | 4.1 | 4 | 4.2 | 0.5 | 0.4 | 0.5 |
| 2016 | 4.5 | 4.5 | 4.6 | 0.5 | 0.4 | 0.5 |
| 2017 | 4.8 | 4.7 | 4.9 | 0.4 | 0.4 | 0.4 |
| 2018 | 4.8 | 4.7 | 4.9 | 0.4 | 0.4 | 0.4 |
| 2019 | 4.9 | 4.8 | 5 | 0.4 | 0.4 | 0.4 |
| 2020 | 6 | 5.8 | 6.1 | 0.4 | 0.4 | 0.4 |

Table 2: AAMRs and 95% Confidence Intervals stratified for gender from 1999-2020.

|  |  | **Type 2 Diabetes** | | | **Type 1 Diabetes** | | |
| --- | --- | --- | --- | --- | --- | --- | --- |
| **Sex** | **Year** | **Age Adjusted Rate** | **Age Adjusted Rate Lower 95% CI** | **Age Adjusted Rate Upper 95% CI** | **Age Adjusted Rate** | **Age Adjusted Rate Lower 95% CI** | **Age Adjusted Rate Upper 95% CI** |
| Female | 1999 | 3.4 | 3.2 | 3.5 | 2 | 2.1 | 2.3 |
| Female | 2000 | 3.7 | 3.6 | 3.8 | 2 | 1.8 | 2 |
| Female | 2001 | 3.6 | 3.5 | 3.8 | 2 | 1.5 | 1.7 |
| Female | 2002 | 3.8 | 3.6 | 3.9 | 1 | 1.3 | 1.5 |
| Female | 2003 | 3.7 | 3.6 | 3.8 | 1 | 1.2 | 1.4 |
| Female | 2004 | 3.6 | 3.5 | 3.7 | 1 | 1 | 1.1 |
| Female | 2005 | 3.7 | 3.6 | 3.9 | 1 | 0.9 | 1.1 |
| Female | 2006 | 3.5 | 3.4 | 3.7 | 1 | 0.8 | 0.9 |
| Female | 2007 | 3.4 | 3.3 | 3.6 | 1 | 0.7 | 0.8 |
| Female | 2008 | 3.4 | 3.3 | 3.6 | 1 | 0.6 | 0.7 |
| Female | 2009 | 3.3 | 3.2 | 3.4 | 1 | 0.6 | 0.7 |
| Female | 2010 | 3.1 | 3 | 3.2 | 1 | 0.5 | 0.6 |
| Female | 2011 | 3 | 2.9 | 3.1 | 1 | 0.4 | 0.5 |
| Female | 2012 | 3 | 2.9 | 3.1 | 0 | 0.4 | 0.5 |
| Female | 2013 | 2.9 | 2.8 | 3 | 1 | 0.4 | 0.5 |
| Female | 2014 | 2.8 | 2.7 | 2.9 | 0 | 0.3 | 0.4 |
| Female | 2015 | 2.9 | 2.8 | 3 | 0 | 0.3 | 0.4 |
| Female | 2016 | 3.2 | 3.1 | 3.3 | 0 | 0.3 | 0.4 |
| Female | 2017 | 3.4 | 3.3 | 3.6 | 0 | 0.3 | 0.4 |
| Female | 2018 | 3.3 | 3.2 | 3.4 | 0 | 0.3 | 0.3 |
| Female | 2019 | 3.4 | 3.3 | 3.5 | 0 | 0.2 | 0.3 |
| Female | 2020 | 4.2 | 4.1 | 4.3 | 0 | 0.3 | 0.4 |
| Male | 1999 | 5.4 | 5.2 | 5.6 | 3 | 2.5 | 2.7 |
| Male | 2000 | 5.6 | 5.4 | 5.8 | 2 | 2.2 | 2.5 |
| Male | 2001 | 5.6 | 5.4 | 5.8 | 2 | 1.9 | 2.1 |
| Male | 2002 | 6 | 5.8 | 6.2 | 2 | 1.8 | 2 |
| Male | 2003 | 6 | 5.8 | 6.2 | 2 | 1.6 | 1.8 |
| Male | 2004 | 6 | 5.8 | 6.2 | 2 | 1.4 | 1.6 |
| Male | 2005 | 5.9 | 5.8 | 6.1 | 1 | 1.3 | 1.5 |
| Male | 2006 | 5.8 | 5.6 | 5.9 | 1 | 1.1 | 1.3 |
| Male | 2007 | 5.5 | 5.3 | 5.7 | 1 | 1 | 1.1 |
| Male | 2008 | 5.6 | 5.5 | 5.8 | 1 | 1 | 1.1 |
| Male | 2009 | 5.5 | 5.3 | 5.7 | 1 | 0.8 | 0.9 |
| Male | 2010 | 5.5 | 5.3 | 5.6 | 1 | 0.9 | 1 |
| Male | 2011 | 5.2 | 5.1 | 5.4 | 1 | 0.7 | 0.9 |
| Male | 2012 | 5.1 | 4.9 | 5.3 | 1 | 0.6 | 0.8 |
| Male | 2013 | 5.3 | 5.2 | 5.5 | 1 | 0.6 | 0.7 |
| Male | 2014 | 5.2 | 5 | 5.4 | 1 | 0.6 | 0.7 |
| Male | 2015 | 5.5 | 5.4 | 5.7 | 1 | 0.6 | 0.7 |
| Male | 2016 | 6.2 | 6 | 6.3 | 1 | 0.5 | 0.6 |
| Male | 2017 | 6.5 | 6.3 | 6.6 | 1 | 0.5 | 0.6 |
| Male | 2018 | 6.7 | 6.5 | 6.9 | 1 | 0.5 | 0.5 |
| Male | 2019 | 6.7 | 6.5 | 6.8 | 1 | 0.5 | 0.6 |
| Male | 2020 | 8.1 | 7.9 | 8.3 | 1 | 0.5 | 0.6 |

Table 3: AAMRs and 95% Confidence Intervals for Metropolitan vs. Non-metropolitan areas from 1999-2020.

|  |  | **Type 2 Diabetes** | | | **Type 1 Diabetes** | | |
| --- | --- | --- | --- | --- | --- | --- | --- |
| **Notes** | **Year** | **Age Adjusted Rate** | **Age Adjusted Rate Lower 95% CI** | **Age Adjusted Rate Upper 95% CI** | **Age Adjusted Rate** | **Age Adjusted Rate Lower 95% CI** | **Age Adjusted Rate Upper 95% CI** |
| Metropolitan | 1999 | 3.9 | 3.8 | 4 | 2.2 | 2.1 | 2.3 |
| Metropolitan | 2000 | 4.1 | 3.9 | 4.2 | 2 | 1.9 | 2 |
| Metropolitan | 2001 | 4.1 | 4 | 4.2 | 1.6 | 1.5 | 1.7 |
| Metropolitan | 2002 | 4.2 | 4.1 | 4.3 | 1.5 | 1.4 | 1.5 |
| Metropolitan | 2003 | 4.2 | 4.1 | 4.4 | 1.3 | 1.3 | 1.4 |
| Metropolitan | 2004 | 4.2 | 4.1 | 4.3 | 1.2 | 1.1 | 1.3 |
| Metropolitan | 2005 | 4.3 | 4.2 | 4.4 | 1 | 1 | 1.1 |
| Metropolitan | 2006 | 4.1 | 4 | 4.3 | 0.9 | 0.8 | 1 |
| Metropolitan | 2007 | 4 | 3.9 | 4.1 | 0.7 | 0.7 | 0.8 |
| Metropolitan | 2008 | 4.1 | 3.9 | 4.2 | 0.7 | 0.7 | 0.8 |
| Metropolitan | 2009 | 4 | 3.9 | 4.1 | 0.7 | 0.6 | 0.7 |
| Metropolitan | 2010 | 3.9 | 3.8 | 4 | 0.6 | 0.6 | 0.7 |
| Metropolitan | 2011 | 3.6 | 3.5 | 3.7 | 0.5 | 0.5 | 0.6 |
| Metropolitan | 2012 | 3.7 | 3.6 | 3.8 | 0.5 | 0.4 | 0.5 |
| Metropolitan | 2013 | 3.7 | 3.6 | 3.8 | 0.5 | 0.5 | 0.5 |
| Metropolitan | 2014 | 3.6 | 3.5 | 3.7 | 0.5 | 0.4 | 0.5 |
| Metropolitan | 2015 | 3.9 | 3.8 | 4 | 0.4 | 0.4 | 0.4 |
| Metropolitan | 2016 | 4.3 | 4.2 | 4.4 | 0.4 | 0.4 | 0.5 |
| Metropolitan | 2017 | 4.5 | 4.4 | 4.6 | 0.3 | 0.3 | 0.4 |
| Metropolitan | 2018 | 4.6 | 4.5 | 4.7 | 0.4 | 0.3 | 0.4 |
| Metropolitan | 2019 | 4.6 | 4.5 | 4.7 | 0.3 | 0.3 | 0.4 |
| Metropolitan | 2020 | 5.7 | 5.5 | 5.8 | 0.4 | 0.3 | 0.4 |
| Non-metropolitan | 1999 | 5.6 | 5.4 | 5.9 | 3.1 | 2.9 | 3.3 |
| Non-metropolitan | 2000 | 6.1 | 5.9 | 6.4 | 2.7 | 2.5 | 2.9 |
| Non-metropolitan | 2001 | 6 | 5.7 | 6.3 | 2.5 | 2.3 | 2.7 |
| Non-metropolitan | 2002 | 6.7 | 6.4 | 7.1 | 2.2 | 2 | 2.4 |
| Non-metropolitan | 2003 | 6.4 | 6.1 | 6.7 | 2 | 1.8 | 2.2 |
| Non-metropolitan | 2004 | 6.3 | 6 | 6.6 | 1.8 | 1.6 | 1.9 |
| Non-metropolitan | 2005 | 6.4 | 6.1 | 6.7 | 1.8 | 1.6 | 2 |
| Non-metropolitan | 2006 | 6.1 | 5.8 | 6.4 | 1.5 | 1.3 | 1.6 |
| Non-metropolitan | 2007 | 5.8 | 5.5 | 6.1 | 1.4 | 1.2 | 1.5 |
| Non-metropolitan | 2008 | 5.8 | 5.5 | 6.1 | 1.3 | 1.1 | 1.4 |
| Non-metropolitan | 2009 | 5.8 | 5.6 | 6.1 | 1.1 | 1 | 1.3 |
| Non-metropolitan | 2010 | 5.4 | 5.2 | 5.7 | 1.1 | 1 | 1.2 |
| Non-metropolitan | 2011 | 5.5 | 5.2 | 5.7 | 1.1 | 1 | 1.3 |
| Non-metropolitan | 2012 | 5.1 | 4.9 | 5.4 | 0.8 | 0.7 | 1 |
| Non-metropolitan | 2013 | 5.1 | 4.9 | 5.4 | 0.9 | 0.8 | 1 |
| Non-metropolitan | 2014 | 5 | 4.7 | 5.2 | 0.9 | 0.8 | 1 |
| Non-metropolitan | 2015 | 5.1 | 4.8 | 5.3 | 0.8 | 0.7 | 0.9 |
| Non-metropolitan | 2016 | 5.6 | 5.3 | 5.9 | 0.7 | 0.6 | 0.8 |
| Non-metropolitan | 2017 | 6.1 | 5.8 | 6.4 | 0.7 | 0.6 | 0.8 |
| Non-metropolitan | 2018 | 6.3 | 6 | 6.5 | 0.6 | 0.5 | 0.7 |
| Non-metropolitan | 2019 | 6.4 | 6.1 | 6.7 | 0.6 | 0.5 | 0.7 |
| Non-metropolitan | 2020 | 7.5 | 7.2 | 7.8 | 0.7 | 0.6 | 0.7 |

Table 4: Age adjusted mortality rates according to census region among type 1 vs type 2 diabetics.

|  |  | **Type 2 Diabetes** | | | **Type 1 Diabetes** | | |
| --- | --- | --- | --- | --- | --- | --- | --- |
| **Census Region** | **Year** | **Age Adjusted Rate** | **Age Adjusted Rate Lower 95% CI** | **Age Adjusted Rate Upper 95% CI** | **Age Adjusted Rate** | **Age Adjusted Rate Lower 95% CI** | **Age Adjusted Rate Upper 95% CI** |
| Census Region 1: Northeast | 1999 | 4 | 3.7 | 4.2 | 2.4 | 2.2 | 2.5 |
| Census Region 1: Northeast | 2000 | 3.9 | 3.6 | 4.1 | 2.1 | 2 | 2.3 |
| Census Region 1: Northeast | 2001 | 3.8 | 3.6 | 4 | 1.7 | 1.5 | 1.8 |
| Census Region 1: Northeast | 2002 | 3.9 | 3.7 | 4.1 | 1.5 | 1.4 | 1.7 |
| Census Region 1: Northeast | 2003 | 3.9 | 3.7 | 4.1 | 1.4 | 1.2 | 1.5 |
| Census Region 1: Northeast | 2004 | 3.8 | 3.6 | 4.1 | 1.3 | 1.2 | 1.4 |
| Census Region 1: Northeast | 2005 | 3.6 | 3.4 | 3.8 | 1.1 | 1 | 1.2 |
| Census Region 1: Northeast | 2006 | 3.3 | 3.1 | 3.5 | 1 | 0.9 | 1.1 |
| Census Region 1: Northeast | 2007 | 3.2 | 3 | 3.4 | 0.8 | 0.7 | 0.9 |
| Census Region 1: Northeast | 2008 | 3.1 | 2.9 | 3.3 | 0.8 | 0.7 | 0.9 |
| Census Region 1: Northeast | 2009 | 2.9 | 2.7 | 3 | 0.7 | 0.6 | 0.8 |
| Census Region 1: Northeast | 2010 | 2.9 | 2.7 | 3.1 | 0.7 | 0.6 | 0.8 |
| Census Region 1: Northeast | 2011 | 2.6 | 2.5 | 2.8 | 0.6 | 0.5 | 0.7 |
| Census Region 1: Northeast | 2012 | 2.7 | 2.6 | 2.9 | 0.5 | 0.4 | 0.6 |
| Census Region 1: Northeast | 2013 | 2.5 | 2.4 | 2.7 | 0.6 | 0.5 | 0.7 |
| Census Region 1: Northeast | 2014 | 2.5 | 2.3 | 2.6 | 0.5 | 0.4 | 0.6 |
| Census Region 1: Northeast | 2015 | 2.5 | 2.3 | 2.6 | 0.4 | 0.3 | 0.5 |
| Census Region 1: Northeast | 2016 | 2.7 | 2.5 | 2.9 | 0.4 | 0.3 | 0.5 |
| Census Region 1: Northeast | 2017 | 2.6 | 2.5 | 2.8 | 0.3 | 0.3 | 0.4 |
| Census Region 1: Northeast | 2018 | 2.8 | 2.7 | 3 | 0.4 | 0.3 | 0.4 |
| Census Region 1: Northeast | 2019 | 2.8 | 2.6 | 3 | 0.3 | 0.3 | 0.4 |
| Census Region 1: Northeast | 2020 | 3.8 | 3.6 | 4 | 0.4 | 0.4 | 0.5 |
| Census Region 2: Midwest | 1999 | 5.3 | 5.1 | 5.6 | 3.1 | 3 | 3.3 |
| Census Region 2: Midwest | 2000 | 5.6 | 5.3 | 5.8 | 2.5 | 2.3 | 2.7 |
| Census Region 2: Midwest | 2001 | 5.6 | 5.3 | 5.8 | 2.1 | 2 | 2.3 |
| Census Region 2: Midwest | 2002 | 5.7 | 5.5 | 6 | 2.1 | 1.9 | 2.2 |
| Census Region 2: Midwest | 2003 | 5.8 | 5.5 | 6 | 1.8 | 1.6 | 1.9 |
| Census Region 2: Midwest | 2004 | 5.5 | 5.2 | 5.7 | 1.5 | 1.4 | 1.6 |
| Census Region 2: Midwest | 2005 | 5.9 | 5.6 | 6.1 | 1.5 | 1.4 | 1.6 |
| Census Region 2: Midwest | 2006 | 5.4 | 5.2 | 5.7 | 1.1 | 1 | 1.2 |
| Census Region 2: Midwest | 2007 | 5.2 | 5 | 5.5 | 1.1 | 1 | 1.2 |
| Census Region 2: Midwest | 2008 | 5.3 | 5.1 | 5.5 | 0.9 | 0.8 | 1 |
| Census Region 2: Midwest | 2009 | 5 | 4.8 | 5.2 | 0.9 | 0.8 | 1 |
| Census Region 2: Midwest | 2010 | 4.6 | 4.4 | 4.8 | 0.8 | 0.7 | 0.9 |
| Census Region 2: Midwest | 2011 | 4.6 | 4.4 | 4.9 | 0.8 | 0.7 | 0.9 |
| Census Region 2: Midwest | 2012 | 4.3 | 4.1 | 4.5 | 0.7 | 0.6 | 0.8 |
| Census Region 2: Midwest | 2013 | 4.4 | 4.2 | 4.6 | 0.7 | 0.6 | 0.8 |
| Census Region 2: Midwest | 2014 | 4.2 | 4 | 4.5 | 0.6 | 0.6 | 0.7 |
| Census Region 2: Midwest | 2015 | 4.4 | 4.2 | 4.6 | 0.5 | 0.5 | 0.6 |
| Census Region 2: Midwest | 2016 | 5 | 4.7 | 5.2 | 0.5 | 0.5 | 0.6 |
| Census Region 2: Midwest | 2017 | 5.3 | 5.1 | 5.5 | 0.5 | 0.5 | 0.6 |
| Census Region 2: Midwest | 2018 | 5.3 | 5.1 | 5.5 | 0.5 | 0.4 | 0.6 |
| Census Region 2: Midwest | 2019 | 5.2 | 5 | 5.5 | 0.5 | 0.4 | 0.6 |
| Census Region 2: Midwest | 2020 | 6.2 | 5.9 | 6.4 | 0.5 | 0.4 | 0.5 |
| Census Region 3: South | 1999 | 4.1 | 3.9 | 4.3 | 2.4 | 2.3 | 2.6 |
| Census Region 3: South | 2000 | 4.6 | 4.4 | 4.8 | 2.2 | 2.1 | 2.4 |
| Census Region 3: South | 2001 | 4.6 | 4.4 | 4.8 | 1.9 | 1.8 | 2.1 |
| Census Region 3: South | 2002 | 4.8 | 4.7 | 5 | 1.7 | 1.6 | 1.8 |
| Census Region 3: South | 2003 | 4.8 | 4.6 | 5 | 1.6 | 1.5 | 1.7 |
| Census Region 3: South | 2004 | 4.6 | 4.5 | 4.8 | 1.3 | 1.2 | 1.4 |
| Census Region 3: South | 2005 | 4.7 | 4.5 | 4.9 | 1.2 | 1.1 | 1.3 |
| Census Region 3: South | 2006 | 4.6 | 4.4 | 4.7 | 1.1 | 1 | 1.2 |
| Census Region 3: South | 2007 | 4.2 | 4 | 4.4 | 0.9 | 0.8 | 1 |
| Census Region 3: South | 2008 | 4.1 | 3.9 | 4.2 | 0.9 | 0.8 | 1 |
| Census Region 3: South | 2009 | 4.3 | 4.1 | 4.4 | 0.8 | 0.7 | 0.9 |
| Census Region 3: South | 2010 | 4.1 | 3.9 | 4.3 | 0.8 | 0.7 | 0.8 |
| Census Region 3: South | 2011 | 3.9 | 3.7 | 4 | 0.7 | 0.6 | 0.8 |
| Census Region 3: South | 2012 | 3.7 | 3.6 | 3.9 | 0.5 | 0.5 | 0.6 |
| Census Region 3: South | 2013 | 3.6 | 3.4 | 3.7 | 0.6 | 0.5 | 0.6 |
| Census Region 3: South | 2014 | 3.5 | 3.4 | 3.7 | 0.5 | 0.5 | 0.6 |
| Census Region 3: South | 2015 | 3.6 | 3.5 | 3.8 | 0.5 | 0.4 | 0.5 |
| Census Region 3: South | 2016 | 4.1 | 3.9 | 4.2 | 0.4 | 0.4 | 0.5 |
| Census Region 3: South | 2017 | 4.3 | 4.1 | 4.4 | 0.4 | 0.3 | 0.4 |
| Census Region 3: South | 2018 | 4.3 | 4.1 | 4.4 | 0.4 | 0.3 | 0.4 |
| Census Region 3: South | 2019 | 4.4 | 4.3 | 4.6 | 0.3 | 0.3 | 0.4 |
| Census Region 3: South | 2020 | 5.4 | 5.2 | 5.5 | 0.4 | 0.3 | 0.4 |
| Census Region 4: West | 1999 | 3.4 | 3.2 | 3.6 | 1.5 | 1.3 | 1.6 |
| Census Region 4: West | 2000 | 3.5 | 3.3 | 3.7 | 1.4 | 1.2 | 1.5 |
| Census Region 4: West | 2001 | 3.6 | 3.4 | 3.8 | 1.1 | 1 | 1.3 |
| Census Region 4: West | 2002 | 4 | 3.8 | 4.2 | 1 | 0.9 | 1.2 |
| Census Region 4: West | 2003 | 3.8 | 3.6 | 4 | 1 | 0.9 | 1.1 |
| Census Region 4: West | 2004 | 4.3 | 4 | 4.5 | 1 | 0.9 | 1.1 |
| Census Region 4: West | 2005 | 4.4 | 4.1 | 4.6 | 0.8 | 0.7 | 0.9 |
| Census Region 4: West | 2006 | 4.5 | 4.3 | 4.7 | 0.7 | 0.6 | 0.8 |
| Census Region 4: West | 2007 | 4.7 | 4.5 | 5 | 0.6 | 0.5 | 0.7 |
| Census Region 4: West | 2008 | 5 | 4.8 | 5.3 | 0.7 | 0.6 | 0.8 |
| Census Region 4: West | 2009 | 4.8 | 4.6 | 5.1 | 0.6 | 0.5 | 0.6 |
| Census Region 4: West | 2010 | 5 | 4.7 | 5.2 | 0.6 | 0.5 | 0.7 |
| Census Region 4: West | 2011 | 4.6 | 4.4 | 4.9 | 0.4 | 0.4 | 0.5 |
| Census Region 4: West | 2012 | 4.9 | 4.7 | 5.1 | 0.5 | 0.4 | 0.6 |
| Census Region 4: West | 2013 | 5.4 | 5.2 | 5.6 | 0.5 | 0.4 | 0.5 |
| Census Region 4: West | 2014 | 5.3 | 5.1 | 5.5 | 0.4 | 0.4 | 0.5 |
| Census Region 4: West | 2015 | 5.9 | 5.6 | 6.1 | 0.4 | 0.3 | 0.5 |
| Census Region 4: West | 2016 | 6.4 | 6.2 | 6.6 | 0.4 | 0.3 | 0.4 |
| Census Region 4: West | 2017 | 7 | 6.7 | 7.2 | 0.4 | 0.3 | 0.5 |
| Census Region 4: West | 2018 | 7 | 6.8 | 7.3 | 0.4 | 0.3 | 0.5 |
| Census Region 4: West | 2019 | 6.9 | 6.7 | 7.2 | 0.4 | 0.3 | 0.4 |
| Census Region 4: West | 2020 | 8.5 | 8.2 | 8.7 | 0.4 | 0.3 | 0.5 |

Table 5: AAMRs and 95% Confidence Intervals stratified for race from 1999-2020

|  |  | **Type 2 Diabetes** | | | **Type 1 Diabetes** | | |
| --- | --- | --- | --- | --- | --- | --- | --- |
| **Hispanic Origin** | **Year** | **Age Adjusted Rate** | **Age Adjusted Rate Lower 95% CI** | **Age Adjusted Rate Upper 95% CI** | **Age Adjusted Rate** | **Age Adjusted Rate Lower 95% CI** | **Age Adjusted Rate Upper 95% CI** |
| Hispanic or Latino | 1999 | 4.9 | 4.4 | 5.5 | 2.7 | 2.3 | 3.1 |
| Hispanic or Latino | 2000 | 5.3 | 4.7 | 5.8 | 1.8 | 1.5 | 2.1 |
| Hispanic or Latino | 2001 | 5.3 | 4.7 | 5.8 | 1.4 | 1.1 | 1.6 |
| Hispanic or Latino | 2002 | 5.3 | 4.8 | 5.9 | 1.5 | 1.2 | 1.8 |
| Hispanic or Latino | 2003 | 5.5 | 4.9 | 6 | 1.3 | 1.1 | 1.6 |
| Hispanic or Latino | 2004 | 5.1 | 4.6 | 5.5 | 1 | 0.8 | 1.3 |
| Hispanic or Latino | 2005 | 5.6 | 5.1 | 6.1 | 1.1 | 0.8 | 1.3 |
| Hispanic or Latino | 2006 | 5.7 | 5.2 | 6.2 | 1 | 0.8 | 1.2 |
| Hispanic or Latino | 2007 | 5.6 | 5.1 | 6 | 0.9 | 0.8 | 1.1 |
| Hispanic or Latino | 2008 | 5.3 | 4.9 | 5.8 | 0.8 | 0.6 | 1 |
| Hispanic or Latino | 2009 | 5.6 | 5.2 | 6.1 | 0.8 | 0.6 | 1 |
| Hispanic or Latino | 2010 | 5.9 | 5.4 | 6.3 | 0.8 | 0.6 | 1 |
| Hispanic or Latino | 2011 | 4.8 | 4.4 | 5.2 | 0.5 | 0.4 | 0.7 |
| Hispanic or Latino | 2012 | 5 | 4.6 | 5.4 | 0.6 | 0.5 | 0.7 |
| Hispanic or Latino | 2013 | 5.3 | 4.9 | 5.7 | 0.5 | 0.4 | 0.6 |
| Hispanic or Latino | 2014 | 5.2 | 4.9 | 5.6 | 0.3 | 0.3 | 0.5 |
| Hispanic or Latino | 2015 | 5.5 | 5.1 | 5.9 | 0.4 | 0.3 | 0.5 |
| Hispanic or Latino | 2016 | 5.9 | 5.5 | 6.3 | 0.4 | 0.3 | 0.5 |
| Hispanic or Latino | 2017 | 6.7 | 6.3 | 7.1 | 0.3 | 0.2 | 0.4 |
| Hispanic or Latino | 2018 | 6.4 | 6 | 6.8 | 0.3 | 0.2 | 0.4 |
| Hispanic or Latino | 2019 | 6.7 | 6.3 | 7.1 | 0.3 | 0.2 | 0.4 |
| Hispanic or Latino | 2020 | 9.4 | 9 | 9.8 | 0.3 | 0.2 | 0.3 |
| Not Hispanic or Latino | 1999 | 4.2 | 4.1 | 4.3 | 2.4 | 2.3 | 2.5 |
| Not Hispanic or Latino | 2000 | 4.4 | 4.3 | 4.5 | 2.1 | 2 | 2.2 |
| Not Hispanic or Latino | 2001 | 4.4 | 4.3 | 4.5 | 1.8 | 1.7 | 1.9 |
| Not Hispanic or Latino | 2002 | 4.7 | 4.5 | 4.8 | 1.6 | 1.6 | 1.7 |
| Not Hispanic or Latino | 2003 | 4.6 | 4.5 | 4.7 | 1.5 | 1.4 | 1.5 |
| Not Hispanic or Latino | 2004 | 4.6 | 4.4 | 4.7 | 1.3 | 1.2 | 1.4 |
| Not Hispanic or Latino | 2005 | 4.7 | 4.5 | 4.8 | 1.2 | 1.1 | 1.2 |
| Not Hispanic or Latino | 2006 | 4.4 | 4.3 | 4.5 | 1 | 1 | 1.1 |
| Not Hispanic or Latino | 2007 | 4.3 | 4.2 | 4.4 | 0.8 | 0.8 | 0.9 |
| Not Hispanic or Latino | 2008 | 4.3 | 4.2 | 4.4 | 0.9 | 0.8 | 0.9 |
| Not Hispanic or Latino | 2009 | 4.2 | 4.1 | 4.3 | 0.8 | 0.7 | 0.8 |
| Not Hispanic or Latino | 2010 | 4 | 3.9 | 4.1 | 0.7 | 0.7 | 0.8 |
| Not Hispanic or Latino | 2011 | 3.9 | 3.8 | 4 | 0.7 | 0.6 | 0.7 |
| Not Hispanic or Latino | 2012 | 3.8 | 3.8 | 3.9 | 0.5 | 0.5 | 0.6 |
| Not Hispanic or Latino | 2013 | 3.9 | 3.8 | 4 | 0.6 | 0.5 | 0.6 |
| Not Hispanic or Latino | 2014 | 3.8 | 3.7 | 3.9 | 0.5 | 0.5 | 0.6 |
| Not Hispanic or Latino | 2015 | 3.9 | 3.8 | 4 | 0.5 | 0.4 | 0.5 |
| Not Hispanic or Latino | 2016 | 4.4 | 4.3 | 4.5 | 0.5 | 0.4 | 0.5 |
| Not Hispanic or Latino | 2017 | 4.6 | 4.5 | 4.7 | 0.4 | 0.4 | 0.5 |
| Not Hispanic or Latino | 2018 | 4.7 | 4.6 | 4.8 | 0.4 | 0.4 | 0.4 |
| Not Hispanic or Latino | 2019 | 4.7 | 4.6 | 4.8 | 0.4 | 0.3 | 0.4 |
| Not Hispanic or Latino | 2020 | 5.6 | 5.5 | 5.7 | 0.4 | 0.4 | 0.5 |

Table 6: Age adjusted mortality rates in type 1 vs type 2 diabetics with AMI from 1999-2020 across 50 states.

|  |  | **Type 2 Diabetes** | | | **Type 1 Diabetes** | | |
| --- | --- | --- | --- | --- | --- | --- | --- |
| **State** | **State Code** | **Age Adjusted Rate** | **Age Adjusted Rate Lower 95% CI** | **Age Adjusted Rate Upper 95% CI** | **Age Adjusted Rate** | **Age Adjusted Rate Lower 95% CI** | **Age Adjusted Rate Upper 95% CI** |
| Alabama | 1 | 2.7 | 2.6 | 2.9 | 0.7 | 0.6 | 0.7 |
| Alaska | 2 | 3.3 | 2.8 | 3.8 | 0.5 | 0.3 | 0.7 |
| Arizona | 4 | 3.2 | 3.1 | 3.3 | 0.5 | 0.4 | 0.5 |
| Arkansas | 5 | 4.7 | 4.5 | 4.9 | 1.5 | 1.4 | 1.7 |
| California | 6 | 6.6 | 6.6 | 6.7 | 0.6 | 0.6 | 0.7 |
| Colorado | 8 | 3.3 | 3.1 | 3.4 | 0.5 | 0.5 | 0.6 |
| Connecticut | 9 | 1.7 | 1.6 | 1.9 | 0.5 | 0.4 | 0.6 |
| Delaware | 10 | 4.2 | 3.8 | 4.5 | 1.1 | 0.9 | 1.3 |
| District of Columbia | 11 | 3.2 | 2.7 | 3.6 | 0.7 | 0.5 | 1 |
| Florida | 12 | 3.3 | 3.3 | 3.4 | 0.8 | 0.8 | 0.9 |
| Georgia | 13 | 2 | 1.9 | 2.1 | 0.5 | 0.5 | 0.6 |
| Hawaii | 15 | 3.4 | 3.2 | 3.7 | 0.3 | 0.2 | 0.4 |
| Idaho | 16 | 5.3 | 5 | 5.6 | 1.1 | 1 | 1.3 |
| Illinois | 17 | 4 | 3.9 | 4.1 | 1 | 0.9 | 1 |
| Indiana | 18 | 5.6 | 5.4 | 5.8 | 1.3 | 1.2 | 1.3 |
| Iowa | 19 | 6.3 | 6.1 | 6.5 | 1.2 | 1.1 | 1.3 |
| Kansas | 20 | 4.5 | 4.2 | 4.7 | 1.2 | 1.1 | 1.3 |
| Kentucky | 21 | 6 | 5.8 | 6.2 | 1.1 | 1 | 1.2 |
| Louisiana | 22 | 1.9 | 1.8 | 2 | 0.9 | 0.8 | 1 |
| Maine | 23 | 4.8 | 4.5 | 5.1 | 0.7 | 0.6 | 0.8 |
| Maryland | 24 | 3.9 | 3.8 | 4.1 | 0.8 | 0.8 | 0.9 |
| Massachusetts | 25 | 1.7 | 1.6 | 1.8 | 0.7 | 0.6 | 0.7 |
| Michigan | 26 | 4.1 | 4 | 4.2 | 1 | 1 | 1.1 |
| Minnesota | 27 | 3.7 | 3.5 | 3.8 | 0.6 | 0.6 | 0.7 |
| Mississippi | 28 | 2.9 | 2.7 | 3.1 | 1.2 | 1.1 | 1.3 |
| Missouri | 29 | 5.1 | 4.9 | 5.3 | 1.1 | 1.1 | 1.2 |
| Montana | 30 | 3 | 2.7 | 3.3 | 0.5 | 0.4 | 0.7 |
| Nebraska | 31 | 3.4 | 3.2 | 3.6 | 0.7 | 0.6 | 0.8 |
| Nevada | 32 | 1.4 | 1.2 | 1.5 | 0.3 | 0.2 | 0.4 |
| New Hampshire | 33 | 3.5 | 3.2 | 3.8 | 0.7 | 0.6 | 0.9 |
| New Jersey | 34 | 3.1 | 3 | 3.2 | 0.9 | 0.9 | 1 |
| New Mexico | 35 | 4.1 | 3.9 | 4.4 | 0.7 | 0.6 | 0.8 |
| New York | 36 | 2.4 | 2.4 | 2.5 | 0.7 | 0.6 | 0.7 |
| North Carolina | 37 | 5.2 | 5.1 | 5.4 | 0.9 | 0.8 | 0.9 |
| North Dakota | 38 | 5.6 | 5.1 | 6.1 | 1.5 | 1.2 | 1.7 |
| Ohio | 39 | 7.8 | 7.6 | 7.9 | 1.4 | 1.3 | 1.4 |
| Oklahoma | 40 | 5.4 | 5.2 | 5.6 | 0.9 | 0.8 | 1 |
| Oregon | 41 | 5.2 | 5 | 5.4 | 0.8 | 0.8 | 0.9 |
| Pennsylvania | 42 | 4.8 | 4.7 | 4.9 | 1.3 | 1.3 | 1.4 |
| Rhode Island | 44 | 4.3 | 4 | 4.6 | 1.8 | 1.6 | 2.1 |
| South Carolina | 45 | 3.8 | 3.6 | 3.9 | 0.8 | 0.7 | 0.9 |
| South Dakota | 46 | 6.2 | 5.7 | 6.6 | 1.1 | 0.9 | 1.3 |
| Tennessee | 47 | 7.8 | 7.6 | 8 | 1.6 | 1.5 | 1.7 |
| Texas | 48 | 5.3 | 5.2 | 5.3 | 0.7 | 0.7 | 0.8 |
| Utah | 49 | 3.3 | 3.1 | 3.6 | 0.5 | 0.4 | 0.6 |
| Vermont | 50 | 4.5 | 4 | 4.9 | 0.8 | 0.6 | 1 |
| Virginia | 51 | 3.6 | 3.5 | 3.8 | 0.7 | 0.7 | 0.8 |
| Washington | 53 | 5.6 | 5.4 | 5.7 | 0.9 | 0.8 | 1 |
| West Virginia | 54 | 7.9 | 7.6 | 8.3 | 1.6 | 1.5 | 1.8 |
| Wisconsin | 55 | 5 | 4.9 | 5.2 | 1 | 1 | 1.1 |
| Wyoming | 56 | 5.5 | 5 | 6.1 | 1 | 0.8 | 1.3 |
